# Prognostic Value of Non-Traditional Lipid Indices for In-Hospital Mortality in Patients with Acute Coronary Syndromes

**DOI:** 10.3390/medicina61050846

**Published:** 2025-05-04

**Authors:** Rustem Yilmaz, Kenan Toprak, Ahmet Karagoz, Osman Can Yontar, Melisa Ucar, Halil Ibrahim Kokcu, Berkant Ozturk, Enes Kaya, Mustafa Yilmaz, Ersoy Öz

**Affiliations:** 1 Department of Cardiology, Faculty of Medicine, Samsun University, Samsun 33805, Turkey; drahmetkgz@hotmail.com (A.K.); drcanyontar@gmail.com (O.C.Y.); mmelisaucar@icloud.com (M.U.); halilkokcu314@gmail.com (H.I.K.); berkant52.dr@gmail.com (B.O.); dreneskaya@gmail.com (E.K.); dr.mustafayilmaz@outlook.com.tr (M.Y.); 2 Department of Cardiology, Faculty of Medicine, Harran University, Şanlıurfa 63050, Turkey; kentoprak@hotmail.com; 3 Department of Statistics, Yildiz Technical University, Istanbul 34220, Turkey

**Keywords:** acute coronary syndrome (ACS), non-traditional lipid indices, atherogenic combined index (ACI), Castelli risk indices (CRI-I and CRI-II), in-hospital mortality prediction, prognostic biomarkers

## Abstract

*Background and Objectives*: Acute coronary syndrome (ACS) is a life-threatening cardiovascular condition with high mortality rates, necessitating accurate and early risk assessment to optimize patient outcomes. While traditional lipid markers, such as low-density lipoprotein-cholesterol (LDL-C) and high-density lipoprotein-cholesterol (HDL-C), are widely used, non-traditional lipid indices, including the lipoprotein combined index (LCI), atherogenic index of plasma (AIP), atherogenic index (AI), Castelli risk indices (CRI-I, CRI-II), and atherogenic combined index (ACI) may offer additional prognostic insights by reflecting the underlying atherogenic and inflammatory processes. This study aimed to assess the prognostic value of these non-traditional lipid indices, along with traditional lipid and biochemical markers, for in-hospital mortality in ACS patients. *Materials and Methods*: This retrospective observational study analyzed data from ACS patients admitted to the coronary care unit (CCU) between January 2019 and September 2024. A cohort of 920 patients was divided into survivor (n = 823, 89.46%) and non-survivor (n = 97, 10.54%) groups based on in-hospital mortality outcomes. Demographic, hematological, biochemical, and lipid profile data, including traditional and non-traditional lipid indices, were collected. Separate logistic regression models were developed for each index, adjusting for demographic and clinical variables in order to assess the independent predictive power of each non-traditional lipid index. *Results*: Significant differences were observed between survivor and non-survivor groups in terms of age, c-reactive protein (CRP), white blood cell count (WBC), hemoglobin (HGB), and creatinine levels (all *p*-values < 0.05). While traditional lipid markers, such as LDL-C and HDL-C, showed limited predictive value, non-traditional lipid indices demonstrated stronger associations. The highest Exp (Beta) values were observed for the CRI-II, AI, and CRI-I. An ROC analysis further confirmed that the CRI-II, AI, and CRI-I had the highest AUC values, with pairwise comparisons underscoring the CRI-II’s superior accuracy. These findings suggest that non-traditional lipid indices predict atherogenic risk better than traditional markers alone. *Conclusions*: Non-traditional lipid indices, particularly the CRI-I and II, AI, LCI, ACI, and AIP, were found to be significantly associated with in-hospital mortality in ACS patients. These indices may provide additional prognostic value beyond traditional lipid parameters; however, further prospective studies are needed to confirm their clinical utility. These results underscore the importance of integrating non-traditional lipid indices into routine risk assessments to improve mortality predictions and inform targeted interventions in high-risk ACS patients.

## 1. Introduction

Acute coronary syndrome (ACS) is the most severe clinical manifestation of coronary artery disease (CAD) and remains a leading cause of death worldwide. Despite the widespread use of percutaneous coronary intervention (PCI), which has significantly reduced cardiovascular mortality and complications, the prognosis for ACS patients remains poor [1]. Atherosclerosis, the primary cause of CAD, is a complex process resulting from lipid accumulation in arterial walls, driven by interactions between atherogenic and anti-atherogenic lipoproteins [2,3]. Traditional lipid markers such as LDL-C, HDL-C, and total cholesterol (TC) have been widely used for cardiovascular risk assessment [4]. However, they do not adequately capture the complexity of atherogenic risk. These markers primarily measure absolute lipid levels without considering interactions or the balance between pro- and anti-atherogenic factors. For instance, LDL-C does not differentiate between large, less atherogenic particles and small, dense particles strongly associated with plaque formation and cardiovascular events. Likewise, HDL-C’s protective role varies as its functionality and anti-inflammatory properties are not always reflected by its absolute level. Furthermore, traditional lipid markers do not sufficiently represent the underlying inflammatory and metabolic disturbances contributing to atherosclerosis, such as insulin resistance, oxidative stress, and systemic inflammation. To address these limitations, studies have explored non-traditional lipid profiles.

Currently, several non-traditional lipid indices have been developed to enhance cardiovascular risk assessment by incorporating traditional lipid profiles. These indices include the atherogenic index (AI = non-HDL-C/HDL-C), atherogenic index of plasma (AIP = log10 [TG/HDL-C]), Castelli risk indices I and II (CRI-I = TC/HDL-C, CRI-II = LDL-C/HDL-C), lipoprotein combined index (LCI = TC × TG × LDL-C/HDL-C), and atherogenic combined index (ACI = log10 [TG × non-HDL-C/HDL-C]) [5,6,7,8,9,10]. These indices provide a more comprehensive assessment of atherogenic burden by reflecting the interactions between various lipid fractions and metabolic processes. They are used as predictive markers for conditions such as atherosclerosis, insulin resistance, hypertension, central obesity, the progression of atherosclerosis, and major adverse cardiac and cerebrovascular events (MACCEs) [10,11,12]. These non-traditional indices enhance predictive accuracy for adverse cardiovascular events by accounting for the interaction between pro-atherogenic lipids (LDL-C, non-HDL-C, and TG) and anti-atherogenic lipoproteins (HDL-C). A recent study identified AIP as an independent risk factor for mortality in acute myocardial infarction (AMI) patients without diabetes mellitus (DM) at 12-month follow-up, while the AI, CRI-I, and CRI-II were not independent predictors of in-hospital death or MACCEs during the same period [13]. Accurate estimation of MACCEs and mortality risk in AMI patients is essential for clinical management as high-risk patients may benefit from more intensive interventional and medical treatments [14].

Furthermore, the atherogenic combined index (ACI), which integrates all lipid parameters, was found to be more effective than other indices in predicting the presence and severity of CAD [6]. This suggests that the ACI may be a valuable independent risk factor for in-hospital mortality and MACCEs as it provides a more comprehensive reflection of atherogenic status.

There is increasing interest in non-traditional lipid indices, such as the AIP, CRI-I, CRI-II, LCI, AI, and ACI, as they may better reflect the underlying pathophysiological processes in ACS. By combining various lipid parameters, these indices offer a more comprehensive assessment of atherogenic potential. The primary objective of this study is to evaluate the prognostic value of non-traditional lipid indices (AI, AIP, LCI, CRI-I, CRI-II, and ACI) for predicting in-hospital mortality in ACS patients. By examining their predictive capabilities, we aim to determine whether these indices can enhance risk stratification beyond traditional lipid markers and provide a more effective basis for guiding tailored therapeutic interventions for high-risk patients.

The biological plausibility of evaluating these indices lies in their ability to reflect the balance between atherogenic lipids (e.g., LDL-C, non-HDL-C, and TG) and anti-atherogenic lipoproteins (HDL-C), which collectively contribute to the development and progression of atherosclerosis. Elevated levels of atherogenic lipids promote endothelial dysfunction, plaque formation, and inflammatory responses, all of which are critical factors in the pathogenesis of ACS. Previous studies have demonstrated associations between higher values of non-traditional lipid indices, such as the AIP, CRI-II, and AI, and increased cardiovascular risk and adverse outcomes, particularly in patients with dyslipidemia and systemic inflammation [13]. Additionally, the atherogenic combined index (ACI) has been suggested to better reflect atherogenic status and predict CAD severity compared to other indices, supporting its potential role as a prognostic marker for in-hospital mortality [6]. Therefore, evaluating these indices may provide a more comprehensive assessment of cardiovascular risk, enhancing predictive accuracy and improving clinical decision-making for high-risk ACS patients.

## 2. Materials and Methods

### 2.1. Study Design and Dataset

This investigation adopts a retrospective observational design, encompassing a cohort of patients with acute coronary syndrome who were hospitalized in the coronary care unit (CCU) at our medical institution between January 2019 and September 2024. The cohort was subsequently dichotomized into two subsets based on the presence or absence of the in-hospital mortality in the hospital, totaling 920 individuals (Group 0: survivors (n = 823, 89.46%); Group 1: non-survivors (n = 97, 10.54%)). This study was conducted in accordance with the Declaration of Helsinki and approved by the Clinical Research Ethics Committee of Samsun University (date: 11 September 2024; protocol number: 2024/16/10).

### 2.2. Inclusion and Exclusion Criteria

Patients with a diagnosis of acute coronary syndrome were included in this study. Acute coronary syndrome (ACS) is divided into two main categories: ST-segment elevation myocardial infarction (STEMI) and non-ST-segment elevation myocardial infarction (NSTEMI). Unstable angina pectoris (USAP) is similar to NSTEMI but without the elevation of cardiac markers. Our study included patients with the following criteria according to the 2017 European Society of Cardiology (ESC) guidelines for the management of acute myocardial infarction (AMI) in patients presenting with STEMI and the 2020 ESC guidelines for the management of acute coronary syndrome (ACS) in patients presenting with NSTEMI [12,15,16,17].

The diagnosis of ACS was made using a combination of clinical symptoms, electrocardiographic (ECG) changes, and elevated cardiac biomarkers (troponin I/T) in accordance with international definitions. Exclusion criteria included patients with incomplete lipid panel data, chronic inflammatory or autoimmune diseases, active malignancy, and end-stage renal disease, as these conditions could introduce confounding effects on lipid metabolism and inflammatory markers.

The exclusion criteria used in the study are given below:

(a) Age < 18 years and >80 years;
(b) A hemoglobin value of less than 8 g/dL;
(c) Lipid-lowering therapy;
(d) A mechanical complication of percutaneous coronary intervention;
(e) Severe renal impairment [estimated glomerular filtration rate (eGFR) of 15 mL/min/m^2^];
(f) A history of cancer was detected within the previous year;
(g) Severe liver impairment;
(h) Missing data about the patient.

### 2.3. Data Collection

Data pertaining to laboratory results, echocardiography findings, demographic variables, MI localizations, and comorbidities were extracted from electronic medical records. Hematological and biochemical parameters, including urea (mg/dL), creatinine, TC LDL-C, TG and HDL-C levels, albumin (Alb), c-reactive protein (CRP), aspartate transaminase (AST), alkaline phosphatase (ALT), troponin (Tn), hemoglobin (Hgb) values, and platelet (PLT) and white blood cell (WBC) counts, were ascertained from venous blood samples obtained during initial admission to the coronary care unit.

### 2.4. Non-Traditional Lipid Profile Calculation

Lipid indices in this study were calculated using standardized equations as previously described in the literature. Specifically, CRI-I (TC/HDL-C), CRI-II (LDL-C/HDL-C), AI (non-HDL-C/HDL-C), AIP (log10 [TG/HDL-C]), LCI (TC × TG × LDL-C/HDL-C), and ACI (log10 [TG × non-HDL-C/HDL-C]) were derived using their respective formulas to ensure consistency and reproducibility of results. These indices were computed directly from the lipid profile data obtained from routine biochemical analyses following standard laboratory methodologies.

(a) The lipoprotein combined index (LCI) was obtained with the formula LCI = TC × TG × LDL-C/HDL-C [5].
(b) The atherogenic combined index (ACI) was obtained through the logarithmic transformation of the ratio of TG multiplied by non-HDL-C to HDL-C (ACI = log10 [TG × non-HDL-C/HDL-C]) [6].
(c) The atherogenic index of plasma (AIP) was calculated through the logarithmic transformation of the ratio of TG and HDL-C concentrations (AIP: log10 [TG/HDL-C]) [7].
(d) The atherogenic index (AI), also known as the atherogenic coefficient, was obtained by dividing non-HDL-C by HDL-C values (AI = non-HDL-C/HDL-C) [8].
(e) The Castelli risk index-I (CRI-I) was obtained by dividing TC by HDL-C values (CRI-I = TC/HDL-C), and CRI-II was obtained by dividing LDL-C by HDL-C values (CRI-II = LDL-C/HDL-C) [9].
(f) Non-HDL-C levels were calculated by subtracting HDL-C from TC values (non-HDL-C = TC-HDL-C) [18].

### 2.5. Statistical Analyses

In this study, an independent samples *t*-test or the Mann–Whitney U test was used to determine whether there was a statistically significant difference between the groups. The Chi-square test was applied for categorical parameters. There are seven different binary logistic regression models to investigate the effects of variables on the survivor group. In the first model, demographic, hematological, biochemical, and traditional lipid variables were included. Since including all demographic, hematological, biochemical, traditional, and non-traditional lipid variables in a single model may lead to multicollinearity issues, six additional models were created, each incorporating one non-traditional lipid variable (AI, AIP, LCI, CRI-I, CRI-II, and ACI) along with the demographic, hematological, and biochemical variables. To ensure model stability and interpretability, multicollinearity was assessed using the Variance Inflation Factor (VIF) for each model. Variables with a VIF greater than 10 were considered to exhibit severe collinearity and were removed from the model. Variables with a VIF between 5 and 10 were carefully evaluated based on their clinical relevance and statistical impact before making a final decision. A VIF below 5 was considered acceptable, indicating no serious multicollinearity concerns. Using this approach ensured that the models remained reliable and that the estimated coefficients were stable and interpretable. This approach ensures that the regression coefficients remain stable and that the predictive power of each non-traditional lipid index can be accurately evaluated in separate models. To ensure consistency and comparability across models, each non-traditional lipid variable was standardized before analysis. Standardization addresses potential scaling issues that could affect interpretation and allows for the direct comparison of effects across models, enhancing the interpretability of each non-traditional lipid variable’s impact on mortality risk. The significance of the logistic regression models was assessed through the Omnibus and Hosmer and Lemeshow tests. The Omnibus test evaluates model significance by testing whether all independent variable coefficients are zero; a rejection of the null hypothesis is desired. The Hosmer and Lemeshow test examines the model’s fit by comparing observed and expected values, with the null hypothesis ideally accepted to confirm good fit. The Cox and Snell and Nagelkerke R² values were used to show how well the model explains variance in the dependent variable, with values closer to 1 indicating better explanatory power. The Wald test was applied to assess the significance of individual coefficients, with the null hypothesis stating that the coefficient is zero. Odds ratios (ORs or Exp(B)) were used to interpret the influence of significant coefficients on the dependent variable, indicating how a one-unit increase in an independent variable affects event probability. However, for standardized non-traditional lipid variables, the OR reflects the change in odds for a one standard deviation increase rather than a one-unit increase in the original scale. This ensures comparability across models by mitigating differences in variable scales and enhancing interpretability. An ROC analysis was conducted to assess the discriminative accuracy of non-traditional lipid indices, with AUC values indicating predictive power. Pairwise ROC comparisons using tests like the DeLong test evaluated significant differences between indices, clarifying each index’s contribution to mortality risk prediction. DeLong’s test was performed to compare the AUC values derived from different logistic regression models (Models 2–7), ensuring appropriate application of the test. The test was not conducted within the same model but rather between independent models, each containing a different non-traditional lipid index, to maintain statistical validity. Additionally, to evaluate the clinical relevance and magnitude of observed differences between survivor and non-survivor groups, effect sizes (Cohen’s d) with 95% confidence intervals (CIs) were calculated for key lipid indices.

**Figure 1:**
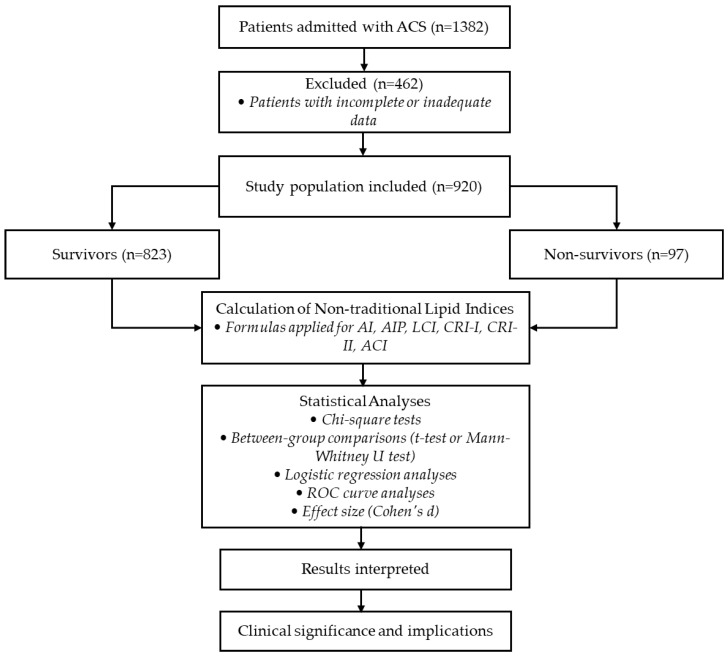
Flowchart of study design and analyses.

A flowchart illustrating the study design, including patient selection, exclusion criteria, calculation of non-traditional lipid indices, and statistical analyses, is presented in Figure 1.

## 3. Results

Table 1 presents a comparison of categorical, demographic, hematological, biochemical, and traditional as well as non-traditional lipid parameters between the survivor and non-survivor groups.

Significant differences were observed between the survivor and non-survivor groups regarding gender (*p* = 0.024), with higher mortality among men. Mortality was significantly higher in patients with diabetes mellitus (DM) compared to those without (*p* < 0.001), and it was also higher in patients with hypertension (*p* = 0.038). Mortality rates did not differ significantly among acute myocardial infarction (AMI) types (*p* = 0.106). However, mortality was significantly higher in patients with ejection fractions (EFs) below 40% compared to those with values above 50% (*p* < 0.001). Additionally, the non-survivor group had a higher average age. There was no statistically significant difference in PLT values between groups. The survivor group had higher HGB levels, whereas the WBC, CRP, and creatinine levels were significantly elevated in the non-survivor group. Among traditional lipid parameters, HDL-C was higher in survivors, while TG, VLDL-C, LDL-C, non-HDL-C, and TC were elevated in non-survivors. All non-traditional lipid parameters (AI, AIP, LCI, CRI-I, CRI-II, and ACI) were also significantly higher in the non-survivor group.

The initial analysis aimed to include all demographic, hematological, biochemical, and traditional lipid variables in Model 1 to comprehensively evaluate their impact on survival outcomes. However, to ensure model stability and reliable coefficient estimation, multicollinearity was assessed using the Variance Inflation Factor (VIF). The results indicate that while most demographic, hematological, and biochemical variables had VIF values between 1 and 2, some of the traditional lipid variables exhibited extremely high collinearity. Specifically, TG (VIF = 71.189), VLDL-C (VIF = 72.714), and TC (VIF = 14.931) exhibited severe multicollinearity, whereas LDL-C (VIF = 9.491) and HDL-C (VIF = 1.954) showed moderate collinearity. To mitigate this issue, TG, VLDL-C, and TC were removed from the model since their VIF values exceeded 10, indicating a high risk of biased coefficient estimation. After excluding these variables, multicollinearity was reassessed, confirming that all remaining variables were within acceptable limits, with the highest VIF value falling below 1.5. LDL-C, which initially had a moderate VIF (9.491), and it was reassessed after the removal of highly collinear lipid variables. Its VIF subsequently dropped below 5, ensuring that its inclusion in the model no longer posed a multicollinearity concern. This ensured that the regression coefficients remained stable and interpretable. The model retained the demographic, hematological, and biochemical parameters and the two remaining traditional lipid variables (HDL-C and LDL-C). Following these adjustments, a logistic regression analysis was conducted. The regression coefficients, odds ratios, and significance levels are presented in Table 2.

The WBC count, HGB, DM, EF_%40–%50_, EF_<%40_, creatinine, CRP, HDL-C, and LDL-C levels and the constant coefficient were found to be significant in Model 1 (Table 2). Higher WBC levels were associated with an increased likelihood of survival, indicating a significant inverse relationship with mortality risk (OR = 1.116, *p* < 0.001). Similarly, higher HGB levels demonstrated a protective effect on survival (OR = 0.837, *p* < 0.05). Patients with diabetes had a significantly lower probability of survival compared to non-diabetic patients (OR = 0.348, *p* < 0.001). Patients with an ejection fraction (EF) between 40 and 50% had a lower survival probability than those with EF levels above 50% (OR = 0.412, *p* < 0.05), while those with EF levels below 40% exhibited an even greater mortality risk (OR = 0.315, *p* < 0.01). Creatinine levels, reflecting renal function, emerged as a significant predictor of survival, with higher creatinine levels being associated with an increased risk of mortality (OR = 1.743, *p* < 0.01). CRP levels were also statistically significant, indicating that elevated CRP values correlated with a higher mortality risk (OR = 1.012, *p* < 0.001). Among traditional lipid parameters, HDL-C was inversely associated with mortality risk, with higher HDL-C levels being linked to greater survival probability (OR = 0.888, *p* < 0.001). In contrast, increased LDL-C levels were associated with a lower likelihood of survival, and the effect of LDL-C on mortality was statistically significant (OR = 1.054, *p* < 0.001).

Multicollinearity was evaluated in all models (Models 2–7) before conducting the logistic regression analysis. In these models, all VIF values were below 1.5, confirming the absence of multicollinearity issues. The logistic regression models and their results are presented in Table 3 and Table 4.

In Model 2, AI emerges as a significant predictor of in-hospital mortality (OR = 3.512, *p* < 0.001), where a one standard deviation increase corresponds to a 251.2% rise in mortality odds, underscoring the impact of atherogenic load. Other significant factors include age, WBC, DM, EF, creatinine, and CRP. High WBC and CRP levels suggest a key role of inflammation, while a reduced EF highlights the importance of cardiac function. Model 3 identifies AIP as a significant predictor (OR = 2.129, *p* < 0.001), with a 112.9% increase in mortality odds per standard deviation rise. This reinforces the link between plasma atherogenicity and mortality. Similarly to Model 2, age, WBC, HGB, DM, EF, creatinine, and CRP remain significant, suggesting a consistent pattern of predictors. Model 4 introduces LCI as a predictor (OR = 2.147, *p* < 0.001), showing a 114.7% increase in mortality odds per standard deviation rise. Its significance suggests that combined lipid profiles offer a broader perspective on atherogenic risk. Age, WBC, HGB, DM, EF, creatinine, and CRP also remain significant, reinforcing the inflammatory process as a crucial factor. In Model 5, the CRI-I is a key predictor (OR = 3.512, *p* < 0.001), with a 251.2% increase in mortality odds per standard deviation rise, emphasizing the influence of the total cholesterol to HDL-C ratio. Age, WBC, DM, EF, creatinine, and CRP are also significant, further highlighting the role of lipid ratios in atherogenic risk assessment. Model 6 identifies the CRI-II as the strongest predictor (OR = 4.149, *p* < 0.001), with a 314.9% increase in mortality odds per standard deviation rise. This result underscores the LDL-C to HDL-C ratio’s predictive power, surpassing total cholesterol ratios. Other significant factors mirror previous models. Finally, Model 7 features ACI (OR = 2.880, *p* < 0.001), where a one-standard deviation increase was linked to an 188.0% rise in mortality odds. This aligns with other atherogenic indices, reinforcing the combined impact of lipid imbalance and inflammation. Significant predictors include age, WBC, HGB, EF, and CRP, highlighting the strong interplay between lipid-related and inflammatory factors. Overall, AI, AIP, LCI, CRI-I, CRI-II, and ACI provide distinct insights into mortality risk, with the CRI-II, CRI-I, and AI being the strongest predictors. Inflammation (WBC and CRP) and cardiac function (EF) consistently play crucial roles in in-hospital mortality among ACS patients.

The significance results of the seven different logistic regression models created to determine the extent to which survivors are affected by other variables can be seen in Table 5. The omnibus test *p*-value was found to be less than 0.001 in all models. The established models are significant, with at least one logistic regression coefficient being different from zero. The *p*-value of the Hosmer and Lemeshow test was found to be greater than 0.05 in all models. There is no significant difference between the observed and expected values in all models. That is, the model data fit is at a sufficient level. When looking at the Cox and Snell and Nagelkerke R square values, Model 1 has the highest value (0.281 and 0.573, respectively). This situation indicates that the relationship between the independent variables in Model 1 and the survivor group is better explained compared to the other models. Model 3 has the lowest R square value (0.192 and 0.392, respectively). The R square coefficients for the other models vary between these two values.

**Figure 2:**
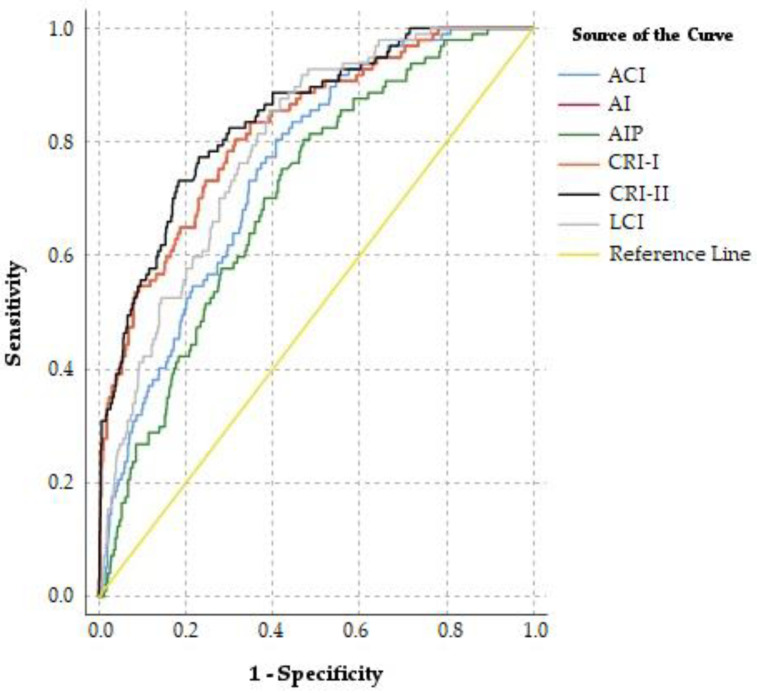
ROC (receiver operating characteristic) curves of non-traditional lipid indices for predicting in-hospital mortality in ACS patients.

In Figure 2, the ROC curves illustrate the predictive accuracy of various non-traditional lipid indices in distinguishing between the survivor and non-survivor groups among ACS patients. Each curve represents the sensitivity and specificity trade-off for an individual index, with a greater area under the curve (AUC) indicating higher discriminative power. Notably, indices such as the CRI-II, AI, and CRI-I exhibit the largest AUC values, highlighting their strong predictive utility for in-hospital mortality. In contrast, indices with lower AUC values, like the AIP and ACI, still provide moderate predictive value but show less distinction between the groups compared to the other indices. These results underscore the overall importance of non-traditional lipid profiles in enhancing risk stratification efforts for ACS patients.

The ROC analysis reveals that the CRI-II, AI, and CRI-I are the most predictive non-traditional lipid indices for in-hospital mortality in ACS patients (Table 6). With the highest AUC at 0.842, the CRI-II demonstrates strong discriminative power and the best specificity (0.817) among the indices, underscoring its utility as a mortality predictor. The AI and CRI-I follow closely, each with an AUC of 0.822, combining solid sensitivity (0.804) and moderate specificity (0.687), suggesting that these indices also effectively capture mortality risk. The LCI, with an AUC of 0.793 and high sensitivity (0.856), shows promise, especially as an early risk indicator. Although the ACI and AIP have slightly lower AUC values, their moderate discriminative power reinforces the overall importance of non-traditional lipid indices in improving risk stratification for ACS patients.

The pairwise comparison of AUC values in Table 7 reveals significant differences among several non-traditional lipid indices. The AI shows no statistically significant differences with the CRI-I but differs from the CRI-II (*p* = 0.033), indicating a slightly lower predictive accuracy compared to the CRI-II. Significant differences were observed between the AIP and most other indices, including the AI, CRI-I, CRI-II, and LCI, underscoring AIP’s relatively lower discriminative capacity for in-hospital mortality. Additionally, while the LCI and CRI-I did not differ significantly, a notable distinction was found between the LCI and CRI-II (*p* = 0.018), suggesting CRI-II’s superior predictive value. The ACI also differed significantly from the CRI-II, CRI-I, and AI, indicating some variability in discriminative power among the indices. These findings indicate that certain indices, such as the CRI-II and CRI-I, are more aligned in predictive value, whereas the AIP shows comparatively lower discriminative power, though still valuable. While the CRI-II demonstrated a statistically significant improvement over the AI (*p* = 0.033), the absolute difference in AUC was only 0.020. Although this difference reached statistical significance, it remains relatively small in a clinical context. In practical applications, an AUC improvement of this magnitude may not necessarily lead to better patient stratification or improved decision-making. Since all models demonstrated reasonably high AUC values (ranging from 0.705 to 0.842), the overall predictive power of these indices remains strong. However, future research should investigate whether these differences translate into meaningful improvements in clinical outcomes by utilizing Net Reclassification Improvement (NRI) or Integrated Discrimination Improvement (IDI) metrics.

To assess the robustness of our findings, we calculated the effect sizes (Cohen’s d) along with their corresponding 95% confidence intervals (CIs) for key non-traditional lipid indices between the survivor and non-survivor groups. Table 8 summarizes these results clearly.

These findings indicate that the CRI-II had the largest effect size (d = 1.714, CI: 1.490–1.939), thus providing the strongest discriminative power for predicting in-hospital mortality in ACS patients. Similarly, the AI and CRI-I also demonstrated large effect sizes (both d = 1.574, CI: 1.351–1.796), highlighting their robust prognostic capabilities. The LCI (d = 1.021, CI: 0.800–1.231) and ACI (d = 0.906, CI: 0.678–1.138) indicated substantial but relatively lower predictive capacities, whereas the AIP exhibited a medium-to-large effect size (d = 0.693, CI: 0.480–0.906). These effect sizes and their associated confidence intervals underline the clinical relevance and robustness of non-traditional lipid indices as valuable tools for improved risk stratification beyond traditional lipid parameters.

## 4. Discussion

This study provides valuable insights into the predictive power of non-traditional lipid indices in assessing in-hospital mortality in patients with ACS. In comparing the survivor and non-survivor groups, a range of demographic, hematologic, biochemical, and lipid parameters demonstrated statistically significant differences, reflecting the complex interplay of atherogenic, inflammatory, and metabolic factors that contribute to ACS outcomes. These findings suggest that non-traditional lipid indices could enhance risk stratification in ACS patients. However, given the retrospective nature of this study, these results should be interpreted with caution, and additional research is necessary to validate their role in clinical decision-making.

In line with the established literature, demographic factors such as the male gender, diabetes mellitus, and hypertension were strongly associated with mortality risk, confirming their role as major cardiovascular risk factors in ACS [19,20,21]. Furthermore, a lower ejection fraction (EF) was correlated with an increased in-hospital mortality risk, consistent with prior studies indicating that reduced left ventricular function is a strong prognostic marker in ACS patients [22]. This aligns with the body of evidence linking impaired EF with adverse outcomes, emphasizing the need for careful monitoring and management of patients with compromised cardiac function. Inflammatory and biochemical markers, particularly WBC, CRP, and creatinine levels, were significantly higher in the non-survivor group. These findings highlight the role of systemic inflammation and organ dysfunction in ACS mortality as inflammation contributes to plaque instability and subsequent ischemic events. Elevated CRP and WBC levels, in particular, indicate heightened inflammatory activity, which is increasingly recognized as a key driver of adverse cardiovascular events [23]. Creatinine, as an indicator of renal function, reflects the burden of end-organ damage, which has been shown to compound mortality risk in ACS patients [24]. Thus, these markers reinforce the prognostic importance of inflammation and organ dysfunction in short-term outcomes.

Traditional lipid parameters further demonstrated notable differences between the survivor and non-survivor groups, with HDL-C levels being significantly higher in survivors, and TG, LDL-C, non-HDL-C, and TC levels were increased in non-survivors. These results align with established knowledge of lipid-driven atherosclerosis, where elevated LDL-C and TG levels are recognized as atherogenic, while higher HDL-C levels offer protective cardiovascular effects [25,26]. However, while traditional lipid markers have predictive value, they often fall short in capturing the full spectrum of atherogenic and inflammatory processes involved in ACS, highlighting the need for more nuanced lipid indices.

Additionally, evidence suggests that even when LDL-C levels are within optimal ranges due to lipid-lowering therapy, residual cardiovascular risk remains. This phenomenon, known as “residual risk”, highlights the limitations of relying solely on LDL-C for risk stratification. Non-traditional lipid indices, by incorporating multiple lipid parameters, offer enhanced prognostic value by capturing residual risk factors that may not be evident through traditional markers alone. In our study, the CRI-II, AI, and CRI-I exhibited stronger associations with in-hospital mortality compared to traditional lipid markers, underscoring their potential utility in refining risk assessment in ACS patients.

Thus, the failure of traditional lipid markers to fully encapsulate the multifaceted nature of atherosclerosis and cardiovascular risk justifies the need for exploring non-traditional lipid indices, which provide a more holistic and clinically relevant approach to mortality prediction in ACS patients.

Non-traditional lipid indices reflect the metabolism or interactions between various lipid fractions. They can be used as predictive indices of atherosclerosis formation, insulin resistance, hypertension, central obesity, the progression of atherosclerosis, and the occurrence of major cardiac and cerebrovascular events (MACCEs) in clinical studies [10,11,12]. Only a few studies have evaluated the predictive value of atherogenic indices for the occurrence of MACCEs and in-hospital mortality in patients after AMI. In a study, the AI, AIP, and LCI were found to be inadequate in predicting the occurrence of MACEs and all-cause mortality at a 1-year follow-up in NSTEMI patients over 60 years of age [27]. Another study evaluated the ratio of CRI-I, CRI-II, and triglycerides to high-density lipoprotein cholesterol in NSTEMI patients and revealed similar results [28]. The above indices were used to determine the risk of MACEs or all-cause mortality in NSTEMI patients and do not reflect the severity of CAD in these patient groups. In another study, the AIP was found to be an independent risk factor for death in patients with acute myocardial infarction (AMI) without diabetes mellitus (DM) at a 12-month follow-up. However, the AI, CRI-I, and CRI-II indices were not found to be independent risk factors for in-hospital death or MACCEs at a 12-month follow-up [13]. Three studies did not fully reflect high-risk patient populations, and therefore, the results may be statistically insignificant. Our study examined a large group of patients with STEMI and NSTEMI and all cardiovascular risk factors.

In this context, non-traditional lipid indices, especially the Castelli risk index II (CRI-II), demonstrated superior performance as mortality predictors, offering a more nuanced and comprehensive assessment of atherogenic risk. Unlike traditional lipid parameters, non-traditional lipid indices incorporate ratios that better reflect the balance between pro-atherogenic and anti-atherogenic lipid particles. The CRI-II, defined as the ratio of LDL-C to HDL-C, showed the highest predictive value among all non-traditional indices, indicating its strong association with in-hospital mortality. This finding is particularly significant as the CRI-II effectively encapsulates both atherogenic burden (through LDL-C) and protective lipid fractions (through HDL-C), making it a robust measure of overall lipid-mediated cardiovascular risk.

The logistic regression models built in this study further illustrate the predictive capacity of non-traditional lipid indices. Each model demonstrated that non-traditional lipid markers, particularly the CRI-II, AIP, and AI, provided significant explanatory power beyond traditional lipid parameters. Since the non-traditional lipid variables were standardized before analysis, the odds ratios (ORs) should be interpreted in terms of one standard deviation increases rather than one-unit increases in their original scale. This approach ensures comparability across models and allows for a clearer understanding of the relative impact of each lipid index on mortality risk. For every one standard deviation increase in the CRI-II, the odds of mortality increased substantially, highlighting its strong association with adverse outcomes in ACS patients. This suggests that the CRI-II not only reflects high levels of atherogenic lipids but also reduces anti-atherogenic protection, emphasizing its role in identifying patients at a higher mortality risk due to an imbalance in their lipid profiles. An ROC analysis further validated the discriminative power of the CRI-II as it achieved the highest area under the curve (AUC) among non-traditional lipid indices. In pairwise comparisons, the CRI-II exhibited superior accuracy compared to other indices, including the AI and CRI-I, reinforcing its potential clinical utility as a key non-traditional marker for mortality risk stratification in ACS patients. Its high AUC value suggests that the CRI-II can reliably distinguish between high-risk and low-risk patients, supporting its role as a robust prognostic tool.

In addition to logistic regression and ROC analyses, we calculated effect sizes (Cohen’s d) along with their 95% confidence intervals to further clarify the magnitude and clinical significance of differences in lipid indices between survivors and non-survivors. These analyses demonstrated that the CRI-II exhibited the largest effect size, highlighting its robustness as a predictor for in-hospital mortality in ACS patients. Similarly, the AI and CRI-I showed strong clinical relevance with large effect sizes, further emphasizing their prognostic utility. The consistency between the logistic regression findings, ROC analysis results, and effect size estimations strengthens the validity and clinical applicability of non-traditional lipid indices in mortality risk stratification.

While other non-traditional indices, such as the AI and CRI-I, also demonstrated strong predictive capacity, they did not match the performance of the CRI-II. Nevertheless, indices like the AIP, LCI, and ACI, though associated with moderate AUC values, contributed additional insights into the atherogenic load and vascular risk. Collectively, these findings support the integration of non-traditional lipid indices with traditional profiles to enhance risk stratification in ACS. Non-traditional indices, by incorporating lipid ratios and reflecting the interplay between atherogenic and protective lipids, offer a more comprehensive view of the atherosclerotic process. These results align with recent studies suggesting that non-traditional lipid indices can improve risk prediction for cardiovascular events and mortality by capturing subtler aspects of lipid metabolism and vascular inflammation. Elevated levels of the CRI-II, AI, and AIP may reflect increased small, dense LDL particles and other lipoprotein subclasses associated with a heightened inflammatory state, which are not readily discernible through traditional lipid measures alone. By integrating non-traditional indices, clinicians may gain a more complete picture of lipid-mediated risk and be better equipped to personalize therapeutic approaches.

In our study, we focused on evaluating the prognostic value of non-traditional lipid indices, including the CRI-II, AI, AIP, LCI, and ACI, in predicting in-hospital mortality. These indices provide a refined assessment of atherogenic risk by integrating multiple lipid parameters beyond traditional lipid markers, capturing aspects such as lipoprotein particle composition and metabolic dysregulation. Previous research has demonstrated that lipid indices, particularly the AIP and CRI-II, correlate with adverse cardiovascular outcomes; however, direct comparisons with established ACS risk scores were beyond the scope of this study. We acknowledge the importance of assessing how these indices compare to scoring systems such as GRACE and TIMI in predictive accuracy. Future research could involve integrating lipid indices into existing risk models or performing comparative analyses to determine whether non-traditional lipid indices enhance risk stratification beyond conventional scoring systems. Such an approach could help refine risk assessment strategies and potentially improve the early identification of high-risk ACS patients. Consequently, these indices should not be considered as an alternative to current risk scoring systems such as GRACE and TIMI, but they can substitute traditional lipid parameters used in the calculation of these scores.

In our study, patients receiving lipid-lowering therapy at the time of admission were excluded to minimize the potential confounding effects of these agents on lipid parameters and atherogenic indices. Statins and other lipid-lowering therapies can significantly alter lipid profiles, potentially affecting the predictive value of non-traditional lipid indices in ACS patients. Our aim in excluding patients undergoing such therapy was to assess the intrinsic prognostic role of these indices without the modifying influence of lipid-lowering interventions.

Although statistically significant differences in AUC values were observed among certain indices, the clinical relevance of these findings should be interpreted with caution. For example, while the CRI-II outperformed the AI with a *p*-value of 0.033, the AUC difference was only 0.020. While statistically significant, this small difference may not be substantial enough to alter clinical decision-making or patient management. Given that all evaluated indices had relatively high AUC values (ranging from 0.705 to 0.842), the overall discriminative ability of these models remains strong, and small variations should be considered in the broader context of clinical applicability. To further assess whether these differences have a meaningful clinical impact, additional measures such as Net Reclassification Improvement (NRI) or Integrated Discrimination Improvement (IDI) could be considered in future studies. These metrics may provide deeper insights into whether these indices improve patient stratification and lead to better clinical outcomes.

## 5. Limitations

Our study has several limitations. The retrospective observational design limits causal inference and may introduce selection bias despite rigorous adjustment for confounding factors. Being a single-center study, our findings might not be generalizable to broader patient populations, potentially affecting external validity. Additionally, the retrospective nature of the study could have resulted in incomplete or inaccurate data collection, potentially affecting the robustness of our conclusions. Future prospective studies with larger, multicenter cohorts are warranted to externally validate our findings and elucidate the mechanistic underpinnings linking non-traditional lipid indices to clinical outcomes in ACS. Finally, although we provided effect sizes with confidence intervals to enhance clinical interpretability, external validation in different patient cohorts remains necessary to confirm the generalizability and practical utility of these non-traditional lipid indices.

Furthermore, as non-traditional lipid indices were standardized before analysis, the odds ratios in our logistic regression models should be interpreted in terms of one standard deviation increases rather than one-unit increases in their original scale. While this approach enhances comparability across models, it may limit direct clinical translation to raw lipid index values. Future research should explore alternative modeling approaches that allow for both standardization and clinically intuitive interpretations of effect sizes.

We acknowledge that factors such as the door-to-balloon time, first medical contact time (FCT), and specific medications (including antiplatelet and antithrombotic agents) can significantly influence patient outcomes and may act as potential confounders in our analysis of non-traditional lipid indices. In our study, we primarily focused on the prognostic value of atherogenic indices in ACS patients independent of immediate procedural and pharmacological interventions. However, we recognize that these time-sensitive variables play a crucial role in in-hospital mortality and should ideally be included in predictive models to ensure a comprehensive risk assessment. While our dataset includes general information on treatment strategies (e.g., percutaneous coronary intervention vs. medical management), we acknowledge that detailed analyses of the door-to-balloon time, FCT, specific medication use, and the distribution of treatment modalities (PCI, bypass surgery, or medical therapy) were not systematically available due to the retrospective nature of our study. Likewise, as coronary angiography reports could not be obtained for all patients, it was not possible to accurately determine and classify the extent of coronary artery involvement (i.e., single-vessel vs. multi-vessel disease). The absence of these detailed treatment data may limit the comprehensiveness of our analysis.

## 6. Conclusions

This study demonstrates that non-traditional lipid indices, including the CRI-II, AI, CRI-I, LCI, ACI, and, AIP, serve as strong predictors of in-hospital mortality in patients with acute coronary syndrome (ACS). Notably, the CRI-II, CRI-I, and AI exhibited the highest odds ratios, underscoring their importance in risk stratification for in-hospital mortality. The significant associations observed between elevated levels of these indices and increased mortality highlight the need to incorporate non-traditional lipid assessments in clinical evaluations, particularly in patients with elevated risk.

## Figures and Tables

**Table 1:** Comparison of parameters (categorical, demographic, hematological, and biochemical data and traditional and non-traditional lipid profiles) between survivor and non-survivor groups.

Variable		Survivor N = 823 (89.46%)	Non-Survivor N = 97 (10.54%)	*p*-Value
*Demographic* *–Categorical*		N (%)	N (%)	
GENDER	Female	239 (29)	39 (40.2)	0.024 *
	Male	584 (71)	58 (59.8)	
DM	Yes	287 (34.9)	63 (64.9)	<0.001 ***
	No	536 (65.1)	34 (35.1)	
HT	Yes	514 (62.5)	71 (73.2)	0.038 *
	No	309 (37.5)	26 (26.8)	
AMI	A. Ant. MI	135 (16.4)	24 (24.8)	0.106
	A. Inf. MI	169 (20.5)	20 (20.6)	
	Non-STEMI	519 (63.1)	53 (54.6)	
EF (%)	>50	506 (61.5)	32 (33.0)	<0.001 ***
	40–50	204 (24.8)	26 (26.8)	
	<40	113 (13.7)	39 (40.2)	
*Demographic*		Mean ± SD	Mean ± SD	
AGE		62.09 ± 11.32	66.60 ± 9.05	<0.001 ***
*Hematological and Biochemical Data*				
HGB (g/dL)		13.34 ± 1.96	12.41 ± 2.03	<0.001 ***
PLT (10^9^/L)		250.12 ± 71.58	264.97 ± 94.24	0.175
WBC (10^9^/L)		10.71 ± 4.61	14.61 ± 6.11	<0.001 ***
CRP (mg/dL)		22.16 ± 36.71	87.27 ± 107.41	<0.001 ***
CREATININ (mg/dL)		1.03 ± 0.55	1.39 ± 0.46	<0.001 ***
*Traditional Lipid Profiles*				
TG (mg/dL)		174.32 ± 102.91	211.11 ± 89.43	<0.001 ***
HDL-C (mg/dL)		41.82 ± 9.15	36.41 ± 8.57	<0.001 ***
VLDL-C (mg/dL)		34.85 ± 20.38	42.18 ± 17.95	<0.001 ***
LDL-C (mg/dL)		126.16 ± 30.85	158.87 ± 28.37	<0.001 ***
TC (mg/dL)		201.01 ± 40.38	234.41 ± 37.47	<0.001 ***
Non-HDL-C (mg/dL)		159.19 ± 36.72	198 ± 36.25	<0.001 ***
*Non-Traditional Lipid Profiles*				
AI		3.94 ± 1.06	5.74 ± 1.70	<0.001 ***
AIP		0.57 ± 0.25	0.74 ± 0.20	<0.001 ***
LCI (×10^3^)		120.15 ± 116.40	245.08 ± 169.63	<0.001 ***
CRI-I		4.94 ± 1.06	6.74 ± 1.70	<0.001 ***
CRI-II		3.1 ± 0.80	4.59 ± 1.32	<0.001 ***
ACI		2.76 ± 0.3	3.03 ± 0.25	<0.001 ***

SD: standard deviation; DM: diabetes mellitus; HT: hypertension; AMI: acute myocardial infarction; A.Ant.MI: acute anterior myocardial infarction; A.Inf.MI: acute inferior myocardial infarction; EF: ejection fraction; HGB: hemoglobin; PLT: platelet; WBC: white blood cell; CRP: C-reactive protein; TG: triglyceride; HDL-C: high-density lipoprotein cholesterol; VLDL-C: very-low-density lipoprotein cholesterol; LDL-C: low-density lipoprotein cholesterol; TC: total cholesterol; AI: atherogenic index; AIP: atherogenic index of plasma; LCI: lipoprotein combined index; CRI: Castelli risk index; ACI: atherogenic combined index. *: Difference is significant at 95% confidence level (two-tailed); ***: difference is significant at 99.9% confidence level (two-tailed).

**Table 2:** Results of binary logistic regression for Model 1.

Variable	Wald Statistics	*p*-Value	Exp(Beta)
Age	2.725	0.099	1.028
Gender_male_	0.627	0.429	1.340
WBC	13.560	<0.001 ***	1.116
HGB	4.221	0.040 *	0.837
PLT	0.503	0.478	0.999
DM_yes_	11.078	0.001 ***	0.348
HT_yes_	0.118	0.731	1.124
AMI_non-STEMI_	1.549	0.213	0.599
AMI_A.INF.MI_	0.017	0.895	1.065
EF_%40–%50_	5.624	0.018 *	0.412
EF_<%40_	8.023	0.005 **	0.315
Creatinin	7.324	0.007 **	1.743
CRP	24.940	<0.001 ***	1.012
HDL-C	36.695	<0.001 ***	0.888
LDL-C	79.662	<0.001 ***	1.054
Constant	6.451	0.011 **	0.005

DM: diabetes mellitus; HT: hypertension; AMI: acute myocardial infarction; A.Inf.MI: acute inferior myocardial infarction; EF: ejection fraction; HGB: hemoglobin; PLT: platelet; WBC: white blood cell; CRP: C-reactive protein; HDL-C: high-density lipoprotein cholesterol; LDL-C: low-density lipoprotein cholesterol. ***: Significance at 99.9% confidence level; **: significance at 99% confidence level; *: significance at 95% confidence level.

**Table 3:** Results of binary logistic regression for Model 2, Model 3, and Model 4.

Variable	Model 2			Model 3			Model 4		
	Wald Statistics	*p*-Value	OR	Wald Statistics	*p*-Value	OR	Wald Statistics	*p*-Value	OR
Age	5.609	0.018 *	1.039	4.448	0.035 *	1.031	5.726	0.017 *	1.037
Gender_male_	1.752	0.186	1.573	0.998	0.318	1.358	0.211	0.646	1.159
WBC	13.420	<0.001 ***	1.108	15.091	<0.001 ***	1.106	16.435	<0.001 ***	1.118
HGB	2.586	0.108	0.881	3.921	0.048 *	0.864	7.102	0.008 **	0.815
PLT	0.762	0.383	0.998	0.125	0.724	0.999	0.208	0.648	0.999
DM_yes_	6.953	0.008 **	0.447	5.583	0.018 *	0.521	6.534	0.011 *	0.486
HT_yes_	0.160	0.689	1.139	0.012	0.914	0.969	0.011	0.916	1.033
AMI_non-STEMI_	1.572	0.210	0.598	2.588	0.108	0.560	2.489	0.115	0.553
AMI_A.INF.MI_	0.000	0.991	0.995	0.107	0.744	0.872	0.015	0.902	1.055
EF_%40–%50_	5.779	0.016 *	0.423	12.142	<0.001 ***	0.323	11.301	0.001 **	0.323
EF_<%40_	6.389	0.011 *	0.378	8.618	0.003 **	0.367	9.073	0.003 **	0.340
Creatinin	4.089	0.043 *	1.481	4.772	0.029 *	1.474	5.145	0.023 *	1.497
CRP	21.192	<0.001 ***	1.010	25.757	<0.001 ***	1.009	27.764	<0.001 ***	1.010
AI	77.598	<0.001 ***	3.512	-	-	-	-	-	-
AIP	-	-	-	25.542	<0.001 ***	2.129	-	-	-
LCI	-	-	-	-	-	-	54.190	<0.001 ***	2.147
Constant	4.864	0.027 *	0.017	3.288	0.070	0.048	2.459	0.117	0.064

DM: diabetes mellitus; HT: hypertension; AMI: acute myocardial infarction; A.Inf.MI: acute inferior myocardial infarction; EF: ejection fraction; HGB: hemoglobin; PLT: platelet; WBC: white blood cell; CRP: C-reactive protein; AI: atherogenic index; AIP: atherogenic index of plasma; LCI: lipoprotein combined index. ***: Significance at 99.9% confidence level, **: significance at 99% confidence level, *: significance at 95% confidence level.

**Table 4:** Results of binary logistic regression for Model 5, Model 6, and Model 7.

Variable	Model 5			Model 6			Model 7		
	Wald Statistics	*p*-Value	OR	Wald Statistics	*p*-Value	OR	Wald Statistics	*p*-Value	OR
Age	5.609	0.018 *	1.039	3.157	0.076	1.029	5.687	0.017 *	1.037
Gender_male_	1.752	0.186	1.573	2.166	0.141	1.688	0.396	0.529	1.220
WBC	13.420	<0.001 ***	1.108	13.561	<0.001 ***	1.112	14.554	<0.001 ***	1.109
HGB	2.586	0.108	0.881	1.841	0.175	0.891	6.649	0.010 *	0.823
PLT	0.762	0.383	0.998	1.038	0.308	0.998	0.176	0.675	0.999
DM_yes_	6.953	0.008 **	0.447	10.534	0.001 **	0.361	3.593	0.058	0.582
HT_yes_	0.160	0.689	1.139	0.144	0.704	1.136	0.003	0.955	1.017
AMI_non-STEMI_	1.572	0.210	0.598	1.320	0.251	0.621	3.284	0.070	0.511
AMI_A.INF.MI_	0.000	0.991	0.995	0.006	0.939	1.037	0.098	0.754	0.874
EF_%40–%50_	5.779	0.016 *	0.423	3.981	0.046 *	0.480	13.033	<0.001 ***	0.296
EF_<%40_	6.389	0.011 *	0.378	5.885	0.015 *	0.380	9.302	0.002 **	0.340
Creatinin	4.089	0.043 *	1.481	5.954	0.015 *	1.642	3.624	0.057	1.423
CRP	21.192	<0.001 ***	1.010	21.607	<0.001 ***	1.010	25.886	<0.001 ***	1.010
CRI-I	77.598	<0.001 ***	3.512	-	-	-	-	-	-
CRI-II	-	-	-	82.056	<0.001 ***	4.149	-	-	-
ACI	-	-	-	-	-	-	47.619	<0.001 ***	2.880
Constant	4.864	0.027 *	0.017	3.931	0.047 *	0.021	2.705	0.100	0.059

DM: diabetes mellitus; HT: hypertension; AMI: acute myocardial infarction; A.Inf.MI: acute inferior myocardial infarction; EF: ejection fraction; HGB: hemoglobin; PLT: platelet; WBC: white blood cell; CRP: C-reactive protein; CRI: Castelli risk index; ACI: atherogenic combined index. ***: Significance at 99.9% confidence level, **: significance at 99% confidence level, *: significance at 95% confidence level.

**Table 5:** The significance results of the models.

Model	Omnibus Test *p*-Value	Hosmer and Lemeshow Test *p*-Value	Cox and Snell R Square	Nagelkerke R Square
Model 1	<0.001 ***	0.668	0.281	0.573
Model 2	<0.001 ***	0.909	0.256	0.522
Model 3	<0.001 ***	0.741	0.192	0.392
Model 4	<0.001 ***	0.648	0.215	0.440
Model 5	<0.001 ***	0.909	0.256	0.522
Model 6	<0.001 ***	0.284	0.271	0.552
Model 7	<0.001 ***	0.758	0.216	0.441

***: The significance at a 99.9% confidence level.

**Table 6:** Predictive performance of non-traditional lipid indices for in-hospital mortality in ACS patients.

Variables	AUC	*p*-Value	Cut-Off	Sensitivity	Specificity
AI	0.822	<0.001 ***	4.342	0.804	0.687
AIP	0.705	<0.001 ***	0.581	0.804	0.530
LCI (×10^3^)	0.793	<0.001 ***	107.27	0.856	0.608
CRI-I	0.822	<0.001 ***	5.343	0.804	0.687
CRI-II	0.842	<0.001 ***	3.763	0.732	0.817
ACI	0.754	<0.001 ***	2.832	0.804	0.593

AI: atherogenic index; AIP: atherogenic index of plasma; LCI: lipoprotein combined index; CRI: Castelli risk index; ACI: atherogenic combined index; AUC: area under curve. ***: Significance at 99.9% confidence level.

**Table 7:** Pairwise comparison of AUC values among non-traditional lipid indices for predicting in-hospital mortality in ACS patients.

Pairs	*p*-Value	AUC Difference
AI − AIP	<0.001 ***	0.117
AI − LCI	0.099	0.029
AI − CRI-I	1.000	0.000
AI − CRI-II	0.033 *	−0.020
AI − ACI	<0.001 ***	0.068
AIP − LCI	<0.001 ***	−0.088
AIP − CRI-I	<0.001 ***	−0.117
AIP − CRI-II	<0.001 ***	−0.137
AIP − ACI	<0.001 ***	−0.049
LCI − CRI-I	0.099	−0.029
LCI − CRI-II	0.018 *	−0.049
LCI − ACI	<0.001 ***	0.039
CRI-I − CRI-II	0.033 *	−0.020
CRI-I − ACI	<0.001 ***	0.068
CRI-II − ACI	<0.001 ***	0.088

AI: atherogenic index; AIP: atherogenic index of plasma; LCI: lipoprotein combined index; CRI: castelliCastelli risk index; ACI: atherogenic combined index. ***: Significance at 99.9% confidence level, *: significance at 95% confidence level.

**Table 8:** Effect sizes (Cohen’s d) and 95% confidence intervals (CIs) for non-traditional lipid indices.

Variables	Cohen’s d	95% CI Lower	95% CI Upper
AI	1.574	1.351	1.796
AIP	0.693	0.480	0.906
LCI (×10^3^)	1.021	0.800	1.231
CRI-I	1.574	1.351	1.796
CRI-II	1.714	1.490	1.939
ACI	0.906	0.678	1.138

## Data Availability

The data presented in this study are available upon request from the corresponding author. The data are not publicly available due to ethical considerations and institutional restrictions regarding patient confidentiality.

## References

[1] Eisen A., Giugliano R.P., Braunwald E. (2016). Updates on Acute Coronary Syndrome: A review. JAMA Cardiol..

[2] Falk E. (2006). Pathogenesis of Atherosclerosis. J. Am. Coll. Cardiol..

[3] Rader D.J., Puré E. (2005). Lipoproteins, macrophage function, and atherosclerosis: Beyond the foam cell?. Cell Metab..

[4] Mach F., Baigent C., Catapano A.L., Koskinas K.C., Casula M., Badimon L., Chapman M.J., De Backer G.G., Delgado V., A Ference B. (2020). 2019 ESC/EAS Guidelines for the management of dyslipidaemias: Lipid modification to reduce cardiovascular risk. Eur. Heart J..

[5] Si Y., Liu J., Han C., Wang R., Liu T., Sun L. (2020). The correlation of retinol-binding protein-4 and lipoprotein combine index with the prevalence and diagnosis of acute coronary syndrome. Heart Vessel..

[6] Toprak K., Kaplangöray M., Karataş M., Dursun A., Arğa Y., Tascanov M.B., Biçer A., Demirbağ R. (2024). Atherogenic Combined Index: Validation of a Novel Predictive Lipid Biomarker for the Presence and Severity of Coronary Artery Disease. Arch. Med. Res..

[7] Shen S.-W., Lu Y., Li F., Yang C.-J., Feng Y.-B., Li H.-W., Yao W.-F., Shen Z.-H. (2018). Atherogenic index of plasma is an effective index for estimating abdominal obesity. Lipids Health Dis..

[8] Aouichat S., Chayah M., Bouguerra-Aouichat S., Agil A. (2020). Time-Restricted Feeding Improves Body Weight Gain, Lipid Profiles, and Atherogenic Indices in Cafeteria-Diet-Fed Rats: Role of Browning of Inguinal White Adipose Tissue. Nutrients.

[9] Vargas H.O., Nunes S.O.V., Barbosa D.S., Vargas M.M., Cestari A., Dodd S., Venugopal K., Maes M., Berk M. (2014). Castelli risk indexes 1 and 2 are higher in major depression but other characteristics of the metabolic syndrome are not specific to mood disorders. Life Sci..

[10] Kosmas C.E., Polanco S.R., Bousvarou M.D., Papakonstantinou E.J., Genao E.P., Guzman E., Kostara C.E. (2023). The Triglyceride/High-Density Lipoprotein Cholesterol (TG/HDL-C) Ratio as a Risk Marker for Metabolic Syndrome and Cardiovascular Disease. Diagnostics.

[11] Cheng W., Zhuang J., Chen S. (2022). Dyslipidemia and the Prevalence of Hypertension: A Cross-Sectional Study Based on Chinese Adults Without Type 2 Diabetes Mellitus. Front. Cardiovasc. Med..

[12] Ibanez B., James S., Agewall S., Antunes M.J., Bucciarelli-Ducci C., Bueno H., Caforio A.L., Crea F., Goudevenos J.A., Halvorsen S. (2018). 2017 ESC Guidelines for the management of acute myocardial infarction in patients presenting with ST-segment elevation: The Task Force for the management of acute myocardial infarction in patients presenting with ST-segment elevation of the European Society of Cardiology (ESC). Eur. Heart J..

[13] Rokicka D., Hudzik B., Wróbel M., Stołtny T., Stołtny D., Nowowiejska-Wiewióra A., Rokicka S., Gąsior M., Strojek K. (2024). Prognostic value of novel atherogenic indices in patients with acute myocardial infarction with and without type 2 diabetes. J. Diabetes Complicat..

[14] Deng C.-J., Yan J., Zheng Y.-Y., Wu T.-T., Pan Y., Hou X.-G., Wang S.-F., Sirajidin S., Aimaitijiang M., Xie X. (2023). Effectiveness of lipid-lowering therapy on mortality and major adverse cardiovascular event outcomes in patients undergoing percutaneous coronary intervention: A network meta-analysis of randomised controlled trials. BMJ Open.

[15] Thygesen K., Alpert J.S., Jaffe A.S., Simoons M.L., Chaitman B.R., White H.D. (2012). Third universal definition of myocardial infarction. Circulation.

[16] Roffi M., Patrono C., Collet J.-P., Mueller C., Valgimigli M., Andreotti F., Bax J.J., A Borger M., Brotons C., Chew D.P. (2016). 2015 ESC Guidelines for the management of acute coronary syndromes in patients presenting without persistent ST-segment elevation: Task Force for the Management of Acute Coronary Syndromes in Patients Presenting without Persistent ST-Segment Elevation of the European Society of Cardiology (ESC). Eur. Heart J..

[17] Thygesen K., Alpert J.S., Jaffe A.S., Chaitman B.R., Bax J.J., Morrow D.A., White H.D. (2018). Fourth universal definition of myocardial infarction (2018). Circulation.

[18] Verbeek R., Hovingh G.K., Boekholdt S.M. (2015). Non-high-density lipoprotein cholesteroll: Current status as cardiovascular marker. Curr. Opin. Lipidol..

[19] Kalyani R.R., Everett B.M., Perreault L., Michos E.D., Lawrence J.M., Casagrande S.S., Herman W.H., Wexler D.J., Cefalu W.T. (2023). Heart Disease and Diabetes. Diabetes in America.

[20] Nkoke C., Jingi A.M., Noubiap J.J., Teuwafeu D., Nkouonlack C., Gobina R., Djibrilla S., Abas A., Dzudie A. (2022). Gender Differences in Cardiovascular Risk Factors, Clinical Presentation, and Outcome of Patients Admitted with a Hypertensive Crisis at the Buea Regional Hospital, Cameroon. Int. J. Hypertens..

[21] Čeponienė I., Žaliaduonytė-Pekšienė D., Gustienė O., Tamošiūnas A., Žaliūnas R. (2014). Association of major cardiovascular risk factors with the development of acute coronary syndrome in Lithuania. Eur. Heart J. Suppl..

[22] Brezinov O.P., Klempfner R., Ben Zekry S., Goldenberg I., Kuperstein R. (2017). Prognostic value of ejection fraction in patients admitted with acute coronary syndrome: A real world study. Medicine.

[23] Amezcua-Castillo E., González-Pacheco H., Martín A.S.-S., Méndez-Ocampo P., Gutierrez-Moctezuma I., Massó F., Sierra-Lara D., Springall R., Rodríguez E., Arias-Mendoza A. (2023). C-Reactive Protein: The Quintessential Marker of Systemic Inflammation in Coronary Artery Disease—Advancing toward Precision Medicine. Biomedicines.

[24] Qi L., Liu H., Cheng L., Cui C., Chen X., Yang S., Cai L. (2021). Impact of Renal Insufficiency on Prognosis of Patients with Acute Coronary Syndrome. Int. J. Gen. Med..

[25] Borén J., Chapman M.J., Krauss R.M., Packard C.J., Bentzon J.F., Binder C.J., Daemen M.J., Demer L.L., Hegele R.A., Nicholls S.J. (2020). Low-density lipoproteins cause atherosclerotic cardiovascular disease: Pathophysiological, genetic, and therapeutic insights: A consensus statement from the European Atherosclerosis Society Consensus Panel. Eur. Heart J..

[26] Nagao M., Nakajima H., Toh R., Hirata K.-I., Ishida T. (2018). Cardioprotective Effects of High-Density Lipoprotein Beyond its Anti-Atherogenic Action. J. Atheroscler. Thromb..

[27] Drwila D., Rostoff P., Nessler J., Konduracka E. (2022). Prognostic significance of atherogenic index of plasma, atherogenic coefficient and lipoprotein combined index among elderly patients with non-ST-segment elevation myocardial infarction in 1-year follow-up. Bratisl. Lek Listy..

[28] Drwiła D., Rostoff P., Nessler J., Konduracka E. (2022). Prognostic value of non-traditional lipid parameters: Castelli Risk Index I, Castelli Risk Index II, and triglycerides to high-density lipoprotein cholesterol ratio among patients with non-ST-segment elevation myocardial infarction during 1-year follow-up. Kardiologiia.

